# The Relation between Obesity and Survival after Surgical Resection of Hepatitis C Virus-Related Hepatocellular Carcinoma

**DOI:** 10.1155/2013/430438

**Published:** 2013-04-24

**Authors:** Hiroki Nishikawa, Akira Arimoto, Tomoko Wakasa, Ryuichi Kita, Toru Kimura, Yukio Osaki

**Affiliations:** ^1^Department of Gastroenterology and Hepatology, Osaka Red Cross Hospital, 5-30 Fudegasaki-cho, Tennoji-ku, Osaka 543-0027, Japan; ^2^Department of Surgery, Osaka Red Cross Hospital, 5-30 Fudegasaki-cho, Tennoji-ku, Osaka 543-0027, Japan; ^3^Department of Pathology, Osaka Red Cross Hospital, 5-30 Fudegasaki-cho, Tennoji-ku, Osaka 543-0027, Japan

## Abstract

*Background and Aims*. We aimed to investigate the relationship between obesity and survival in hepatitis C virus-(HCV-) related hepatocellular carcinoma (HCC) patients who underwent curative surgical resection (SR). *Methods*. A total of 233 patients with HCV-related HCC who underwent curative SR were included. They included 60 patients (25.8%) with a body mass index (BMI) of > 25 kg/m^2^ (obesity group) and 173 patients with a BMI of < 25 kg/m^2^ (control group). Overall survival (OS) and recurrence-free survival (RFS) rates were compared. *Results*. The median follow-up periods were 3.6 years in the obesity group and 3.1 years in the control group. The 1-, 3-, and 5-year cumulative OS rates were 98.3%, 81.0%, and 63.9% in the obesity group and 90.0%, 70.5%, and 50.3% in the control group (*P* = 0.818). The corresponding RFS rates were 70.1%, 27.0%, and 12.0% in the obesity group and 70.1%, 39.0%, and 21.7% in the control group (*P* = 0.124). There were no significant differences between the obesity group and the control group in terms of blood loss during surgery (*P* = 0.899) and surgery-related serious adverse events (*P* = 0.813). *Conclusions*. Obesity itself did not affect survival in patients with HCV-related HCC after curative SR.

## 1. Introduction 

Hepatocellular carcinoma (HCC), which accounts for more than 90% of primary liver cancers, is a major health problem worldwide. It is the fifth most common cancer in men, the seventh most common cancer in women, and the third most common cause of cancer-related death [1–4]. Although hepatitis B virus (HBV) and hepatitis C virus (HCV) infections are both major causes of HCC, HCV-related HCC represents approximately 70% of all HCC cases [5]. 

The prognosis for untreated HCC is generally poor, and the curative therapies for HCC consist of liver transplantation and surgical resection (SR) [1–3]. However, HCC frequently recurs even after curative SR, leading to high mortality, although it only recurs at intrahepatic sites in 68%–96% of patients [6, 7]. Stringent followup of HCC patients and optimal management of HCC recurrence is therefore essential.

Obesity is a major health problem throughout the world [8]. An increasing body of epidemiological evidence indicates that the obesity is associated with increased risk of several cancers of the breast, endometrium, kidney, colon, liver, pancreas, gallbladder, and esophagus [8]. An explanation for the association between obesity and HCC may be that obesity increases the risk of nonalcoholic steatohepatitis (NASH) and HCC can be a late complication of NASH [9]. 

Calle et al. conducted a large prospective study and reported that obesity was associated with significantly increased risk of HCC-related death [8]. However, Park et al. found in their large nationwide survey that a body mass index (BMI) > 25 kg/m^2^ did not lead to a significant increase in mortality in HCC patients [10]. These discordant findings may be explained by the different BMI cutoff values used in the two studies. However, to the best of our knowledge, there are few data regarding the effects of obesity on postoperative clinical outcomes in HCV-related HCC patients who underwent respective surgery [11]. Thus, the objectives of the current study were to examine the effect of obesity on survival rates in HCV-related HCC patients who underwent curative SR.

## 2. Patients and Methods 

### 2.1. Patients

Patients were selected for SR based on assessment of tumor characteristics, liver function, remnant liver volume, and general condition, through discussion with experienced surgeons, radiologists, and physicians. The choice of anatomical or nonanatomical hepatectomy was made based on hepatic functional reserve and tumor location. 

**Figure 1:**
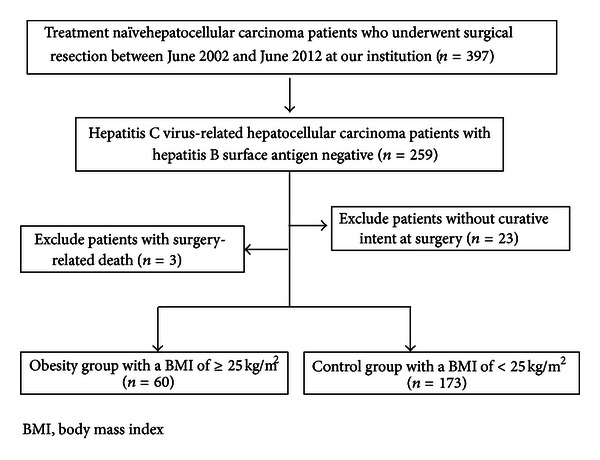
Study profile.

SR was performed in 397 treatment-naive HCC patients at the Department of Surgery, Osaka Red Cross Hospital, Japan, between June 2002 and June 2012. Of these patients, 259 (65.2%) were HCV-related HCC patients negative for the HBV surface antigen (HBsAg) and positive for the HCV antibody (HCVAb). Patients operated on without curative intent (*n* = 23) and with surgery-related death (*n* = 3) were excluded from the study. Curative surgery was defined as the resection of all tumors detectable with imaging modalities. Thus, a total of 233 HCV-related HCC patients were included in the present study (Figure 1). 

Included patients were classified into an obesity group with a BMI of > 25 kg/m^2^ (*n* = 60 [25.8%]; range: 25.0–36.1 kg/m^2^) and a control group with a BMI of < 25 kg/m^2^ (*n* = 173 [74.2%]; range: 14.9–24.9 kg/m^2^). The WHO definition of obesity is BMI > 30 kg/m^2^ [12]. However, in Japan, the proportion of the population with a BMI of > 30 kg/m^2^ has been reported to be no more than 2%-3%, in contrast with the 20%–30% prevalence in Western countries. In Japan, the definition of obesity is proposed to be a BMI of > 25 kg/m^2^ because of an increased incidence of obesity-related disorders in people with a BMI of > 25 kg/m^2^ [13–15]. Overall survival (OS) and recurrence-free survival (RFS) rates were compared between the two groups. A diagnosis of diabetes mellitus was based on past medical history or 75-g oral glucose tolerance test results [16].

This study was approved by the ethics committee of Osaka Red Cross Hospital, Japan, and the need for written informed consent in the current study was waived because the data were analyzed retrospectively and anonymously. Written informed consent was obtained from all patients prior to surgery, and the study protocol complied with all of the provisions of the Declaration of Helsinki. The present study comprised a retrospective analysis of patient records registered in our database, and all treatments were conducted in an open-label manner.

### 2.2. HCC Diagnosis

HCC was diagnosed using abdominal ultrasound and dynamic computed tomography (CT) scans (hyperattenuation during the arterial phase in all or some parts of the tumor and hypoattenuation in the portal-venous phase) and/or magnetic resonance imaging (MRI), based mainly on the recommendations of the American Association for the Study of Liver Diseases [17]. Arterial- and portal-phase dynamic CT images were obtained at approximately 30 and 120 s, respectively, after the injection of the contrast material. HCC stage was determined using the Liver Cancer Study Group of Japan staging system [18]. HCC was confirmed pathologically in resection specimens at surgery. 

### 2.3. Serological Studies

HBsAg was detected using commercial enzyme immunoassay kits (Dainabot, Tokyo, Japan) [19]. HCVAb was assessed using second-generation assays (Dainabot) [20]. Serum HCV RNA levels were tested in 184 (79.0%) of the 233 patients using a competitive reverse transcription-PCR assay. High hepatitis C viral load was defined according to guidelines [19, 21]. 

### 2.4. Followup

Followup after surgery consisted of periodic blood tests and monitoring of tumor markers, including alpha-fetoprotein (AFP) and des-*γ*-carboxy prothrombin (DCP), using chemiluminescent enzyme immunoassays (Lumipulse PIVKAII Eisai; Eisai, Tokyo, Japan). Dynamic CT and/or MRI scans were obtained every 2–4 months after surgery. Chest CT, whole abdominal CT, brain MRI, and bone scintigraphy were performed when extrahepatic HCC recurrence was suspected. 

### 2.5. Statistical Analysis

Data were analyzed using univariate and multivariate analyses. Continuous variables were compared using unpaired *t*-tests, and categorical variables were compared using Fisher's exact tests. Time to recurrence was defined as the interval between each therapy and first confirmed recurrence. For analysis of RFS, the followup ended at the time of first recurrence; other patients were censored at their last follow-up visit or the time of death from any cause without recurrence. For analysis of OS, the followup ended at the time of death from any cause, and the remaining patients were censored at the last follow-up visit. The cumulative OS and RFS rates were calculated using the Kaplan-Meier method and tested using the log-rank test. Factors with a *P* value <0.05 in univariate analysis were subjected to multivariate analysis using the Cox proportional hazards model. These statistical methods were used to estimate the interval from initial treatment. Data were analyzed using SPSS (SPSS Inc., Chicago, IL, USA). Data are expressed as mean ± standard deviation (SD). Values of *P* < 0.05 were considered statistically significant.

## 3. Results

### 3.1. Baseline Characteristics

The baseline characteristics of the patients in the two groups can be found in Table 1. The median observation periods were 3.6 years (range: 0.7–8.4 years) in the obesity group and 3.1 years (range: 0.3–10.9 years) in the control group. The obesity group patients were significantly younger than the control group patients (*P* = 0.013). Total bilirubin (*P* = 0.043) and indocyanine green retention at 15 min (ICGR 15) (*P* = 0.027) were higher, and platelet count (*P* = 0.043) was lower in the obesity group than in the control group, indicating that the obesity group had poorer hepatic function than the control group. However, there were no differences between the groups in the Child-Pugh classification (*P* = 0.795). 

### 3.2. Cumulative OS and RFS Rates

**Figure 2:**
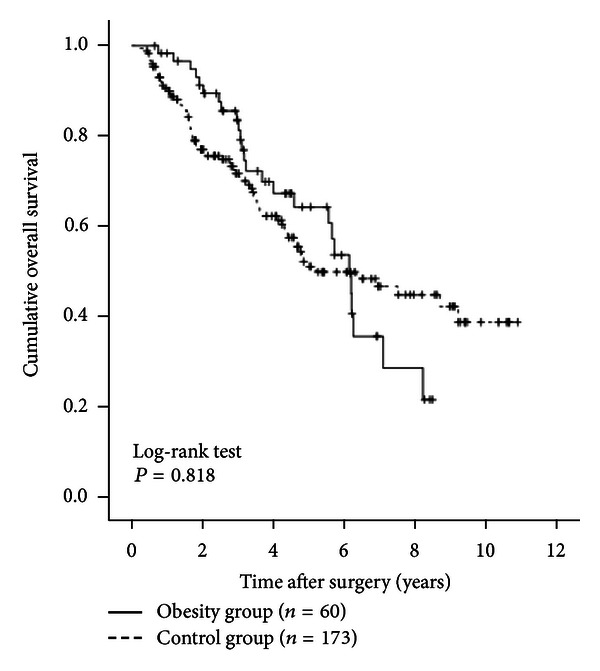
Cumulative overall survival (OS) rates in the obesity group (*n* = 60) and the control group (*n* = 173). The 1-, 3-, and 5-year cumulative OS rates were 98.3%, 81.0%, and 63.9%, respectively, in the obesity group and 90.0%, 70.5%, and 503%, respectively, in the control group (*P* = 0.818).

**Figure 3:**
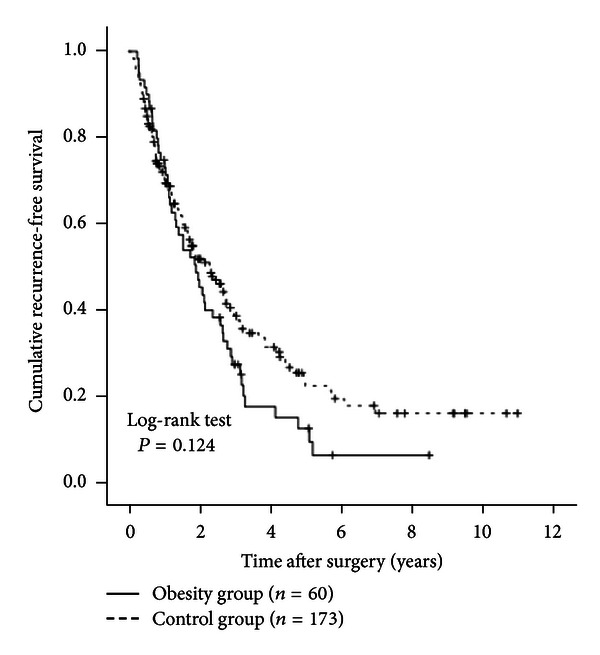
Cumulative recurrence-free survival (RFS) rates in the obesity group (*n* = 60) and the control group (*n* = 173). The 1-, 3-, and 5-year cumulative RFS rates were 70.1%, 27.0%, and 12.0%, respectively, in the obesity group and 70.1%, 39.0%, and 21.7%, respectively, in the control group (*P* = 0.124).

The 1-, 3-, and 5-year cumulative OS rates were 98.3%, 81.0%, and 63.9%, respectively, in the obesity group and 90.0%, 70.5%, and 50.3%, respectively, in the control group (*P* = 0.818) (Figure 2). The 1-, 3-, and 5-year cumulative RFS rates were 70.1%, 27.0%, and 12.0%, respectively, in the obesity group and 70.1%, 39.0%, and 21.7%, respectively, in the control group (*P* = 0.124) (Figure 3).

### 3.3. Univariate and Multivariate Analyses of Factors Contributing to OS

Univariate analysis identified the following factors as significantly associated with OS for all cases (*n* = 233): HCC stage (*P* < 0.001), maximum tumor size > 3.5 cm (*P* = 0.016), tumor number (*P* = 0.005), interferon (IFN) therapy after surgery (*P* = 0.039), ICGR 15 > 13.5% (*P* = 0.038), serum albumin > 3.8 g/dL (*P* < 0.001); AFP > 20 ng/mL (*P* < 0.001), and microscopic vascular invasion (*P* < 0.001) (Table 2). The hazard ratios (HRs) and 95% confidence intervals (CIs) calculated using multivariate analysis for the eight factors that were significant in univariate analysis are detailed in Table 3. Serum albumin > 3.8 g/dL (*P* = 0.002), AFP > 20 ng/mL (*P* < 0.001), and microscopic vascular invasion (*P* = 0.006) were found to be significant predictors linked to OS in multivariate analysis.

### 3.4. Univariate and Multivariate Analyses of Factors Contributing to RFS

Univariate analysis identified the following factors as significantly associated with RFS for all cases (*n* = 233): HCC stage (*P* < 0.001): tumor number (*P* < 0.001): IFN therapy after surgery (*P* = 0.019): aspartate aminotransferase (AST) > 60 IU/L (*P* = 0.042): alanine aminotransferase (ALT) > 50 IU/L (*P* = 0.014): AFP > 20 ng/mL (*P* = 0.010): DCP > 100 mAU/mL (*P* = 0.031): and microscopic vascular invasion (*P* < 0.001) (Table 2). The HRs and 95% CIs calculated using multivariate analysis for the eight factors that were significant in univariate analysis are detailed in Table 4. IFN therapy after surgery (*P* = 0.010) and microscopic vascular invasion (*P* = 0.001) were shown to be significant prognostic factors linked to RFS.

### 3.5. Causes of Death in the Two Groups

Twenty-six patients in the obesity group (43.3%) died during the follow-up period. The causes of death were HCC recurrence in 19 patients and liver failure in seven. Seventy-two patients in the control group (41.6%) died during the follow-up period, and the causes of death were HCC recurrence in 47 patients, liver failure in 14, and miscellaneous causes in eleven. 

### 3.6. HCC Recurrence

Fifty patients in the obesity group (83.3%) and 109 patients in the control group (63.0%) had HCC recurrence during the follow-up period. The patterns of HCC recurrence after surgery in the obesity group were single HCC recurrence in the liver in 21 patients, multiple HCC recurrences in the liver in 27 patients, multiple HCC recurrences in the liver with lung metastases in one patient, and single brain metastasis in one patient. The patterns of HCC recurrence after surgery in the control group were single HCC recurrence in the liver in 44 patients, single HCC recurrence in the liver with portal vein invasion in one patient, multiple HCC recurrences in the liver in 52 patients, multiple HCC recurrences in the liver with portal vein invasion in two patients, multiple HCC recurrences in the liver with lymph node metastases in four patients, multiple HCC recurrences in the liver with lung metastases in two patients, multiple HCC recurrences in the liver with adrenal metastases in one patient, multiple HCC recurrences in the liver with inferior vena cava invasion in one patient, multiple HCC recurrences in the liver with peritoneal dissemination in one patient, and multiple HCC recurrences in the liver with bone metastases in one patient. 

### 3.7. Treatment Methods for HCC Recurrence

Treatment methods for the first HCC recurrence in the obesity group were SR in three patients, radiofrequency ablation (RFA) in 31 patients, transcatheter arterial chemoembolization (TACE) in 12 patients, systemic chemotherapy in one patient, and no specific treatment in three patients. The treatment methods used in the control group were SR in 10 patients, RFA in 46 patients, TACE in 35 patients; PEI in six patients, radiotherapy in three patients, systemic chemotherapy in two patients, and no specific treatment in seven patients.

### 3.8. IFN Therapy after Surgery

Sixteen patients (5 patients (3.5%) in the obesity group and 11 (6.4%) in the control group) received IFN therapy after surgery. They included stage I HCC in two patients, stage II in nine patients, stage III in four patients, and stage IV in one patient. Whether IFN treatment after surgery was performed was mainly decided by the attending physicians. All patients who received IFN therapy had high viral load prior to the therapy, as defined by guidelines [19, 21]. Fourteen patients received peginterferon and ribavirin combination therapy, and two received long-term low-dose IFN maintenance therapy. Seven patients (43.8%) achieved sustained virological response (SVR) as defined by undetectable HCV RNA 24 weeks after completion of IFN treatment. Seven patients (43.8%) had HCC recurrence, and two patients (12.5%) died during the follow-up period. 

### 3.9. Subgroup Analyses According to Age

**Figure 4:**
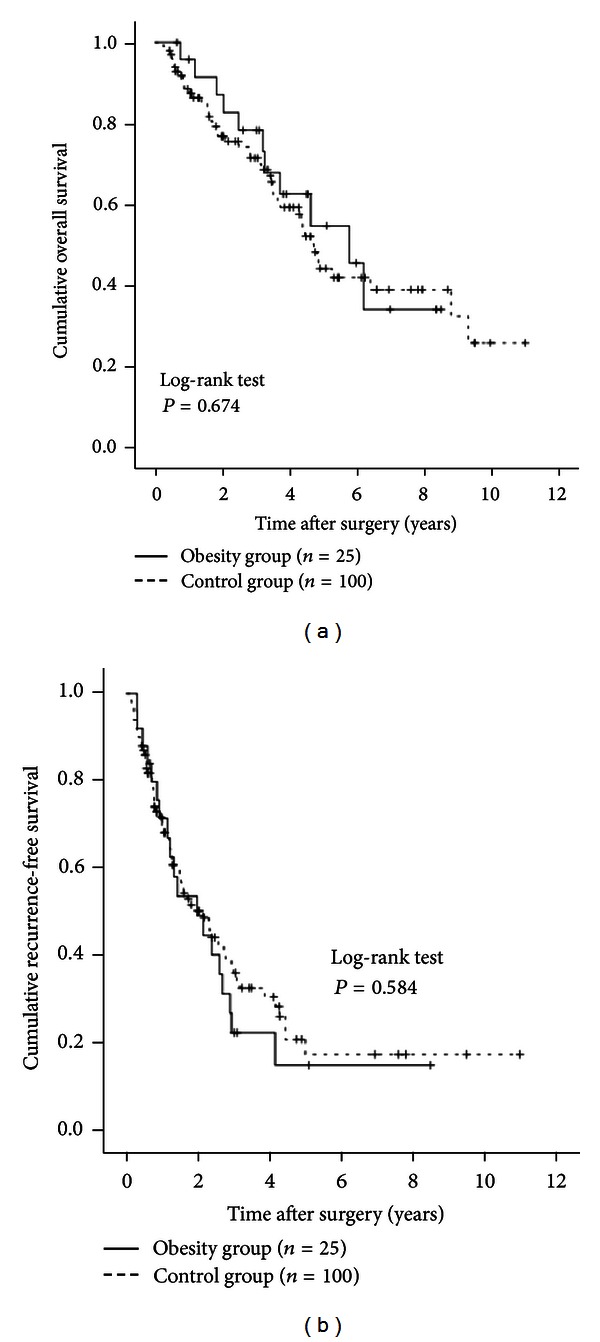
Subgroup analyses in patients aged 70 years or more (*n* = 125). There were 25 patients aged 70 years or more in the obesity group and 100 in the obesity group. There were no significant differences between the two groups in terms of OS (a) (*P* = 0.674) and RFS (b) (*P* = 0.584).

**Figure 5:**
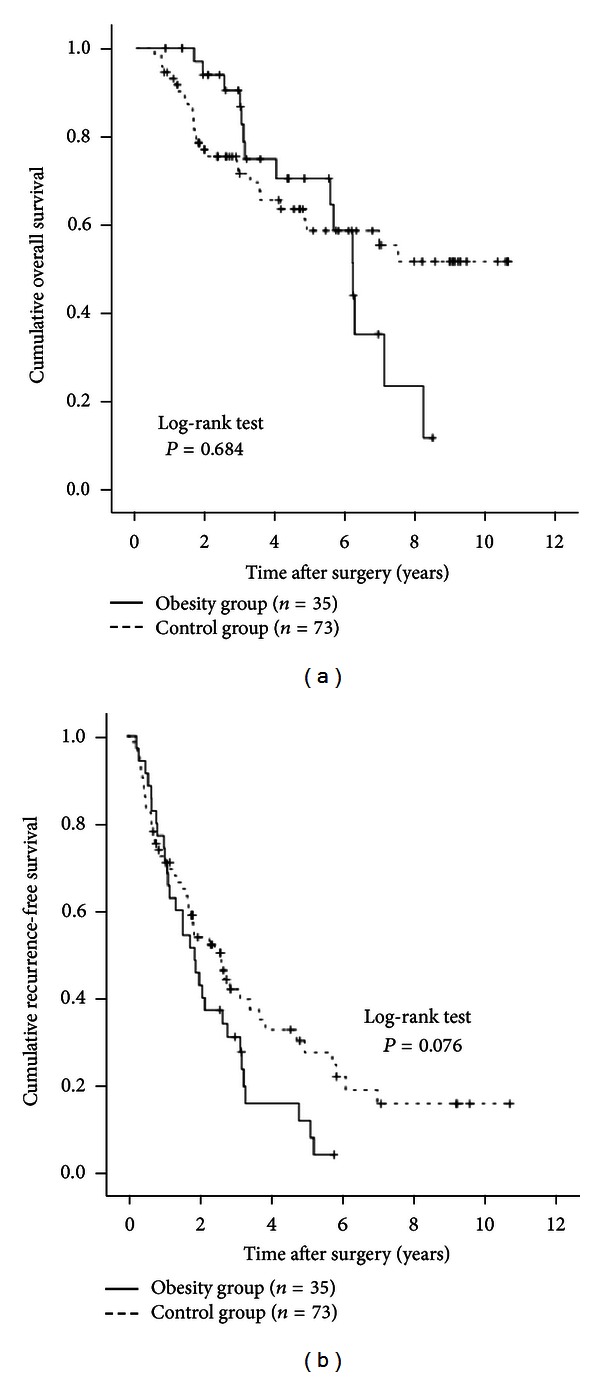
Subgroup analyses in patients aged less than 70 years (*n* = 108). There were 35 patients aged younger than 70 years in the obesity group and 73 in the control group. There were no differences between the two groups in terms of OS (a) (*P* = 0.684). However, the RFS rates in the obesity group tended to be lower than those in the control group, although the difference did not reach significance (b) (*P* = 0.076).

Patients in the obesity group were significantly younger than patients in the control group at the time of diagnosis. We therefore performed subgroup analyses according to age. In patients aged 70 years or more (25 (41.7%) in the obesity group and 100 (57.8%) in the control group), no significant differences were observed in OS (*P* = 0.674) or RFS (*P* = 0.584) between the obesity group and the control group (Figures 4(a) and 4(b)). In patients aged less than 70 years (35 (58.3%) in the obesity group and 73 (42.2%) in the control group), there was no significant difference in OS (*P* = 0.684) between the two groups. RFS rates in the obesity group tended to be lower than those in the control group, although the difference did not reach significance (*P* = 0.076) (Figures 5(a) and 5(b)).

### 3.10. Subgroup Analyses According to the Child-Pugh Classification

**Figure 6:**
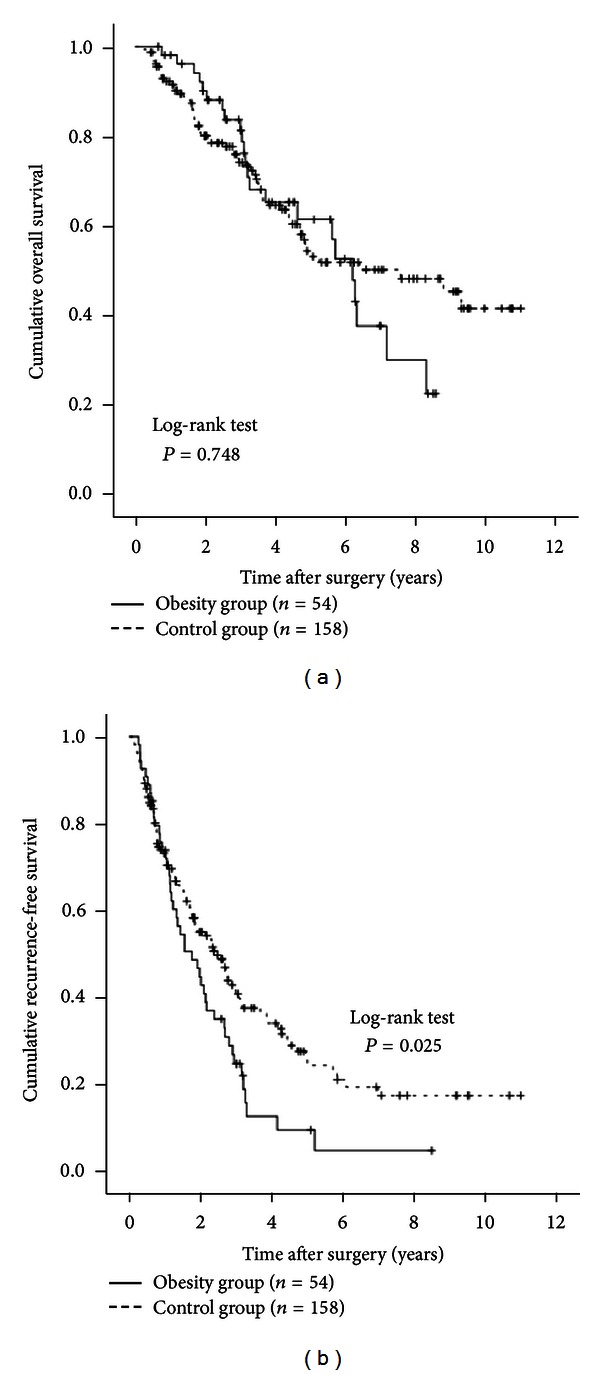
Subgroup analyses in patients with the Child-Pugh grade A (*n* = 212). There were 54 patients with the Child-Pugh grade A in the obesity group and 158 in the control group. In terms of OS (a), there was no significant difference between the two groups (*P* = 0.748). RFS rates were significantly higher in the control group than in the obesity group (b) (*P* = 0.025).

**Figure 7:**
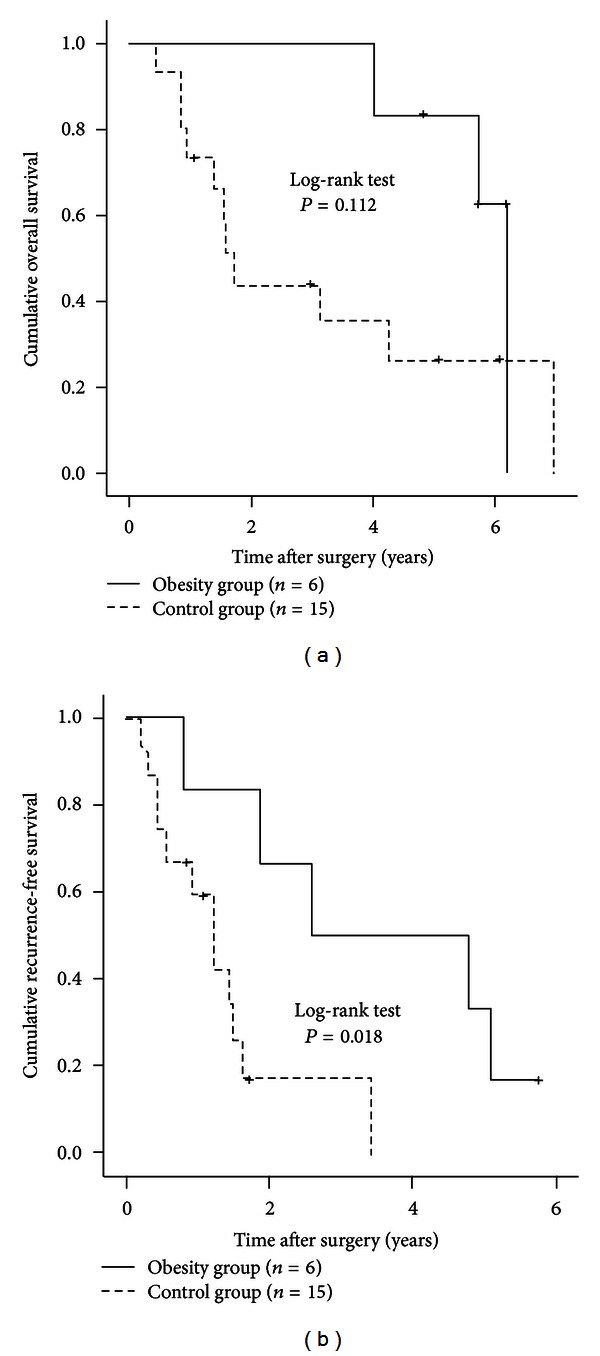
Subgroup analyses in patients with the Child-Pugh grade B (*n* = 21). There were six patients with the Child-Pugh grade B in the obesity group and 15 in the control group. In terms of OS (a), there was no significant difference between the two groups (*P* = 0.112). However, RFS rates in the obesity group were significantly higher than those in the control group (b) (*P* = 0.018).

We performed subgroup analyses in patients with Child-Pugh grade A (54 (90.0%) in the obesity group and 158 (91.3%) in the control group) and patients with the Child-Pugh grade B (6 (10.0%) in the obesity group and 15 (8.7%) in the control group). No significant difference was observed in OS between the two groups in the Child-Pugh A patients (*P* = 0.748, Figure 6(a)). RFS rates were significantly higher in the control group than in the obesity group (*P* = 0.025, Figure 6(b)). In patients with the Child-Pugh grade B, there was no significant difference in OS between the two groups (*P* = 0.112, Figure 7(a)). However, the RFS rates in the obesity group were significantly higher than those in the control group (*P* = 0.018, Figure 7(b)). 

### 3.11. Blood Loss during Surgery and Surgery-Related Serious Adverse Events (SAEs) in the Two Groups

Mean ± SD blood loss during surgery was 816.4 ± 905.5 mL in the obesity group and 799.0 ± 798.9 mL in the control group (*P* = 0.899). Surgery-related SAEs in the obesity group included abscess formation in two patients, bile leakage in one patient, refractory ascites in three patients, and sepsis in one patient. Equivalent complications in the control group included abscess formation in five patients, bile leakage in four patients, refractory ascites in seven patients, aspiration pneumonia in two patients, gastrointestinal bleeding in two patients, perforation of the small intestine in one patient, and acute respiratory distress syndrome in one patient. All these SAEs improved during the same hospitalization period. There were no significant differences between the two groups in terms of SAEs related to surgery (*P* = 0.813). 

### 3.12. Further Analysis According to BMI

**Figure 8:**
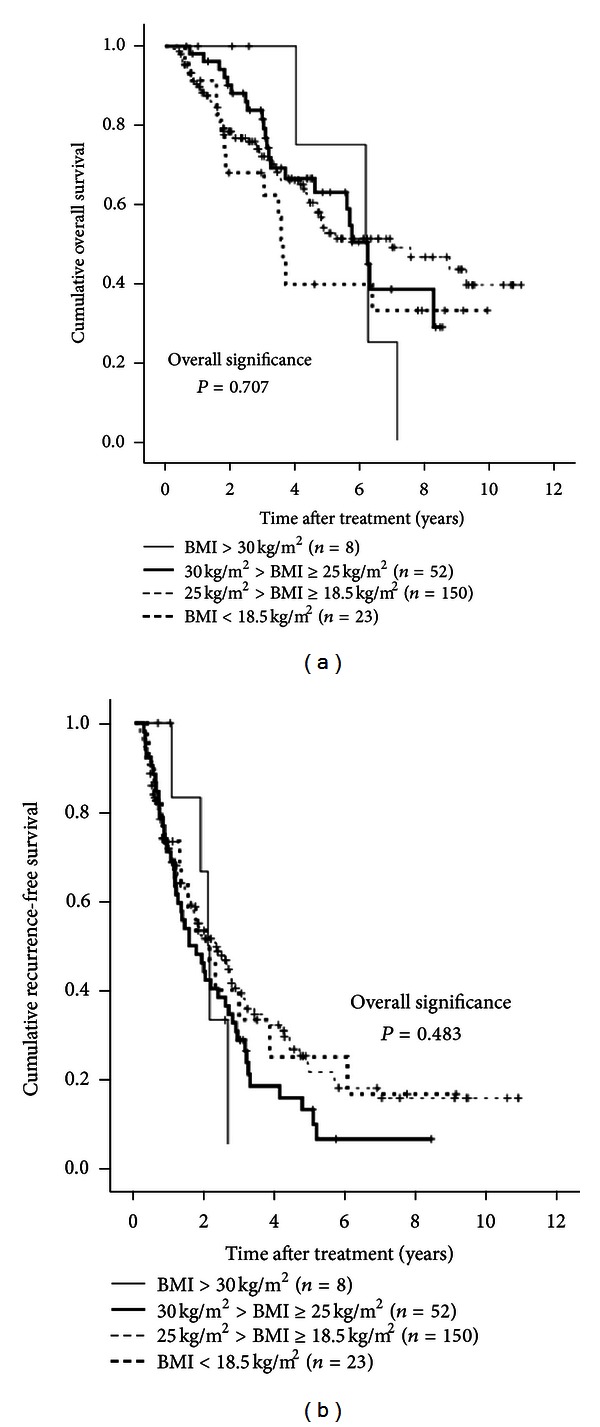
The Kaplan-Meier curves using classification into four groups according to BMI (BMI ≥ 30 kg/m^2^ (*n* = 8), 30 kg/m^2^ > BMI ≥ 25 kg/m^2^ (*n* = 152), 25 kg/m^2^ > BMI ≥ 18.5 kg/m^2^ (*n* = 150), and BMI < 18.5 kg/m^2^ (*n* = 23)). In terms of overall survival (*P* = 0.707) and recurrence-free survival (*P* = 0.483), the overall difference in these four groups did not reach significance ((a) and (b)).

According to the WHO classification, we classified the obese group patients into two groups (patients with a BMI of > 30 kg/m^2^ [*n* = 8] and those with a BMI of < 30 kg/m^2^ [*n* = 52]) [12]. Similarly, we classified the control group patients into two groups (patients with a BMI of > 18.5 kg/m^2^ [*n* = 150] and those with a BMI of < 18.5 kg/m^2^ [*n* = 23]). We compared OS and RFS rates in these four groups. In terms of OS, there was no significant difference in these four groups (overall significance; *P* = 0.707) (Figure 8(a)). In terms of RFS, the difference in the four groups did not also reach significance (overall significance; *P* = 0.483) (Figure 8(b)). 

## 4. Discussion

To the best of our knowledge, few studies have investigated the effects obesity on survival after curative resection for HCV-related HCC [11]. Hence, it remains unclear whether obesity can be a prognostic factor after curative resection for HCV-related HCC. We therefore conducted this comparative observational study. 

In general, obesity increases the risk of various medical problems that can adversely affect clinical outcomes in HCC patients. However, we found that obesity, as defined by BMI ≥ 25 kg/m^2^, was not a significant adverse prognostic factor when all cases were considered. There also were no differences in RFS or OS rates between the two groups in most subgroup analyses. In addition, when we classified our all cases (*n* = 233) into four groups according to BMI (BMI ≥ 30 kg/m^2^, 30 kg/m^2^ > BMI ≥ 25 kg/m^2^, 25 kg/m^2^ > BMI ≥ 18.5 kg/m^2^, and BMI < 18.5 kg/m^2^), the difference in these four groups did not reach significance in terms of both OS and RFS. However, in patients with the Child-Pugh grade A, RFS rates were lower in obese patients than in control patients, while in patients with the Child-Pugh grade B RFS rates were higher in obese patients than in control patients. These results indicate that obesity is not associated with adverse clinical outcomes after curative resection of HCV-related HCC, although, in patients with well-preserved liver function and poor liver function, obesity may be associated with changed RFS after curative SR for HCC. One possible reason for this lack of effect is that only eight patients (3.4%) with a BMI ≥ 30 kg/m^2^, which can adversely affect clinical outcomes, were included in our study. As described above, the proportion of the population with a BMI of ≥ 30 kg/m^2^ in Japan is substantially lower than that in Western countries. 

In the present study, the obesity group patients were significantly younger at time of diagnosis than the control group patients. Akiyama et al. demonstrated that increased BMI is associated with increased risk for early HCC development in patients with HCV infection and the association between earlier HCC development and increased BMI was the result of the hepatic oxidative stress [22], which is in agreement with the results from the present study. Thus, in obese patients with HCV infection, close observation for early HCC occurrence will be needed. 

In baseline characteristics, the obesity group patients had significantly higher total bilirubin levels, lower platelet counts, and higher ICGR 15 levels, indicating that the obesity group had more advanced background liver disease compared with the control group, although no significant difference between the two groups was observed in terms of the Child-Pugh classification. Obesity is linked with hepatic steatosis and adversely affects the progression of chronic HCV liver disease [23–25].

In our multivariate analyses, the presence of microvascular invasion was a significant adverse predictor for both OS and RFS. Microvascular invasion, caused by HCC tumor cells, may provide an important route for intrahepatic metastasis and thus leads to poorer clinical outcomes. Our results are consistent with those of several other studies that identified the presence of microvascular invasion as a significant adverse prognostic factor for HCC patients after surgery [26–28]. Serum AFP level was the strongest adverse predictor for OS but only had marginal significance in terms of RFS. Ikai et al. reported that high pretreatment AFP levels were an adverse prognostic factor for survival [29], which is in agreement with our results. Serum albumin level was also significant predictor linked to OS. HCC patients with cirrhosis and low levels of serum albumin may develop protein-energy malnutrition (PEM) with increased catabolism [30]. In such patients, invasive surgery will lead to increased risk of poor clinical outcomes after SR [31]. In the management of HCC patients who underwent curative SR, clinicians should be alert to not only tumor-related factors but also liver function-related factors. 

In the present study, IFN therapy after surgery was associated with increased RFS rate with an HR of 2.709, although the number of patients who received IFN therapy was small. Singal et al. reported in their meta-analysis that IFN treatment after curative resection of HCV-related HCC prevented HCC recurrence and improved survival [32]. Our results were in agreement with their report, although four (57.1%) out of seven patients who achieved SVR after curative resection for HCC in the present study had HCC recurrence. Close observation for HCC recurrence will therefore be needed even in HCC patients who achieved SVR after curative SR. 

In terms of blood loss during surgery and surgery-related SAEs, no significant difference between the obesity group and the control group was observed, indicating that the obesity did not have an effect on perioperative and postoperative complications. This result is consistent with those of previous reports [11, 33]. 

 This study had several limitations including the retrospective nature of the study and relatively short median observation period in the two groups. However, our study results indicate that the obesity itself does not affect the survival in HCC patients who underwent curative SR. In conclusion, the consideration of obesity may not be necessary for the management of HCC patients who receive curative SR. 

## Figures and Tables

**Table 1:** Baseline characteristics between the obesity group and the control group.

Variables	Obesity group (*n* = 60)	Control group (*n* = 173)	*P* value
Age (years)	67.2 ± 8.1	70.2 ± 8.1	0.013^a^
Gender: male/female	40/20	119/54	0.872^b^
Body mass index (kg/m^2^)	27.6 ± 2.6	21.3 ± 2.4	<0.001^a^
HCC stage			
Stage I/II/III/IV	7/40/11/2	12/45/97/19	0.407^b^
Type of hepatectomy			
Anatomical /nonanatomical	15/45	63/110	0.115^b^
Maximum tumor size (cm)	3.6 ± 1.6	4.2 ± 2.5	0.087^a^
Tumor number, the single/multiple	45/15	117/56	0.331^b^
Child-Pugh classification			
The Child-Pugh A/B	54/6	158/15	0.795^b^
Hepatitis C viral load			
High/low/unknown	39/11/10	109/25/39	0.554^b^
IFN therapy after surgery: yes/no	5/55	11/162	0.767^b^
AST (IU/L)	70.7 ± 42.2	66.5 ± 49.2	0.561^a^
ALT (IU/L)	65.6 ±49.7	59.3 ± 42.0	0.342^a^
Serum albumin (g/dL)	3.7 ± 0.5	3.8 ± 0.5	0.555^a^
Total bilirubin (mg/dL)	0.95 ± 0.47	0.82 ± 0.40	0.043^a^
Prothrombin time (%)	87.1 ± 14.0	87.7 ± 13.4	0.756^a^
Platelets (×10^4^/mm^3^)	11.7 ± 5.0	13.4 ± 5.7	0.043^a^
ICGR 15	17.6 ± 11.0	14.4 ± 9.1	0.027^a^
AFP (ng/mL)	528.6 ± 1870.3	2422.7 ± 13113.3	0.267^a^
DCP (mAU/mL)	1505.3 ± 4975.0	3789.9 ± 16524.2	0.293^a^
Total cholesterol (IU/L)	170.7 ± 35.0	161.7 ± 33.0	0.083^a^
Diabetes mellitus: yes/no	19/41	45/128	0.405^b^

Data are expressed as number or mean ± standard deviation. HCC: hepatocellular carcinoma, IFN: interferon, AST: aspartate aminotransferase, ALT: alanine aminotransferase, ICGR 15: indocyanine green retention at 15 min, AFP: alpha-fetoprotein, DCP: des-*γ*-carboxy prothrombin, ^a^unpaired *t* test, and ^b^Fisher^,^s exact test.

**Table 2:** Univariate analysis contributing to OS and RFS for all cases (*n* = 233).

Variables	*n *	OS	RFS
		*P* value^a^	*P* value^a^
Age ≥ 70 (yes/no)	125/108	0.096	0.779
Gender (male/female)	159/74	0.070	0.445
HCC stages (I, II/III, and IV)	163/70	<0.001	<0.001
Maximum tumor size ≥ 3.5 cm (yes/no)	116/117	0.016	0.071
Tumor number (single/multiple)	162/71	0.005	<0.001
IFN therapy after surgery (yes/no)	16/217	0.039	0.019
Body mass index ≥ 25 kg/m^2^ (yes/no)	60/173	0.818	0.124
Diabetes mellitus (yes/no)	64/169	0.311	0.994
ICGR 15 ≥ 13.5% (yes/no)	115/118	0.038	0.402
Total bilirubin ≥ 0.8 mg/dL (yes/no)	115/118	0.142	0.487
Serum albumin ≥ 3.8 g/dL (yes/no)	128/105	<0.001	0.088
AST ≥ 60 IU/L (yes/no)	110/123	0.090	0.042
ALT ≥ 50 IU/L (yes/no)	113/120	0.261	0.014
Platelets ≥ 12 × 10^4^/mm^3^ (yes/no)	128/105	0.065	0.679
Prothrombin time ≥ 87% (yes/no)	122/111	0.236	0.067
AFP ≥ 20 ng/mL (yes/no)	122/111	<0.001	0.010
DCP ≥ 100 mAU/mL (yes/no)	135/98	0.144	0.031
Total cholesterol ≥ 160 IU/L (yes/no)	109/124	0.476	0.575
Microscopic capsule (yes/no)	185/48	0.630	0.551
Microscopic capsule invasion (yes/no)	140/93	0.215	0.467
Microscopic vascular invasion (yes/no)	73/160	<0.001	<0.001
Microscopic surgical margin (yes/no)	30/203	0.642	0.112

OS: overall survival, RFS: recurrence-free survival, HCC: hepatocellular carcinoma, IFN: interferon, ICGR 15: indocyanine green retention at 15 min, AST: aspartate aminotransferase, ALT: alanine aminotransferase, AFP: alpha-fetoprotein, DCP: des-*γ*-carboxy prothrombin, and ^a^log-rank test.

**Table 3:** Multivariate analysis associated with OS after surgical resection for hepatocellular carcinoma.

Variable	Hazard ratio	95% confidence interval	*P* value^a^
HCC stage			
Stage I or II	1.000		0.069
Stage III or IV	0.423	0.168–1.070	
Maximum tumor size			
≥3.5 cm	0.765	0.502–1.167	0.214
<3.5 cm	1.000		
Tumor number			
Single	1.299	0.520–3.244	0.576
Multiple	1.000		
IFN therapy after surgery			
Yes	3.054	0.745–12.520	0.121
No	1.000		
ICGR 15			
≥13.5%	0.954	0.623–1.460	0.828
<13.5%	1.000		
Serum albumin level			
≥3.8 g/dL	1.981	1.295–3.031	0.002
<3.8 g/dL	1.000		
AFP level			
≥20 ng/mL	0.425	0.274–0.660	<0.001
<20 ng/mL	1.000		
Microscopic vascular invasion			
Yes	0.549	0.359–0.840	0.006
No	1.000		

OS: overall survival, HCC: hepatocellular carcinoma, IFN: interferon, ICGR 15: indocyanine green retention at 15 min, AFP: alpha-fetoprotein, and ^a^The Cox proportional hazard model.

**Table 4:** Multivariate analysis associated with RFS after surgical resection for hepatocellular carcinoma.

Variable	Hazard ratio	95% confidence interval	*P* value^a^
HCC stage			
Stage I or II	1.000		0.766
Stage III or IV	0.900	0.449–1.802	
Tumor number			
Single	1.000		0.115
Multiple	0.573	0.287–1.144	
IFN therapy after surgery			
Yes	2.709	1.264–5.807	0.010
No	1.000		
AST			
≥60 IU/L	1.091	0.651–1.595	0.933
<60 IU/L	1.000		
ALT			
≥50 IU/L	0.770	0.489–1.211	0.257
<50 IU/L	1.000		
AFP			
≥20 ng/mL	0.739	0.522–1.046	0.088
<20 ng/mL	1.000		
DCP			
≥100 mAU/mL	0.846	0.612–1.170	0.313
<100 mAU/mL	1.000		
Microscopic vascular invasion			
Yes	0.560	0.397–0.788	0.001
No	1.000		

RFS: recurrence-free survival, HCC: hepatocellular carcinoma, IFN: interferon, AST: aspartate aminotransferase, ALT: alanine aminotransferase, AFP: alpha-fetoprotein, DCP: des-*γ*-carboxy prothrombin, and ^a^The Cox proportional hazard model.
